# Mobile genetic elements in *Klebsiella pneumoniae*

**DOI:** 10.1128/jb.00012-25

**Published:** 2025-04-29

**Authors:** Ting Pan, Qingrong Li

**Affiliations:** 1 School of Life Sciences and Laboratory Medicine, Kunming Medical University 71240, Kunming, China; University of Notre Dame, Notre Dame, Indiana, USA

**Keywords:** *Klebsiella pneumoniae*, mobile genetic elements, virulence factors, antibiotic resistance

## Abstract

*Klebsiella pneumoniae* is a clinically important pathogenic bacteria that poses a serious threat to human health. In particular, the emergence of hypervirulent and multidrug-resistant *K. pneumoniae* has posed great challenges in clinical anti-infective therapy. In the *K. pneumoniae* genome, mobile genetic elements (MGEs), such as plasmids, prophages, transposons, and insertion sequences, enhance bacterial viability and adaptation by mediating the horizontal transfer of virulence genes, antibiotic resistance genes, and other adaptive genes. This paper reviews the types and characteristics of the main MGEs in *K. pneumoniae*, focusing on their effects on bacterial virulence and antibiotic resistance, with the aim of providing clues for developing infection control measures and new antibacterial drugs.

## INTRODUCTION

*Klebsiella pneumoniae* is a common gram-negative bacillus that is widespread in the natural environment and also found in the intestinal and respiratory tracts of humans and animals. *K. pneumoniae* is an important pathogen of hospital- and community-acquired infections, causing pneumonia, urinary tract infections, sepsis, and other serious infections. Over time, *K. pneumoniae* has evolved significantly in terms of pathogenicity and resistance (1), progressing from classical *K. pneumoniae* (cKP) to hypervirulent *K. pneumoniae* (hvKP) and multidrug-resistant (MDR) *K. pneumoniae*.

**Fig 1:**
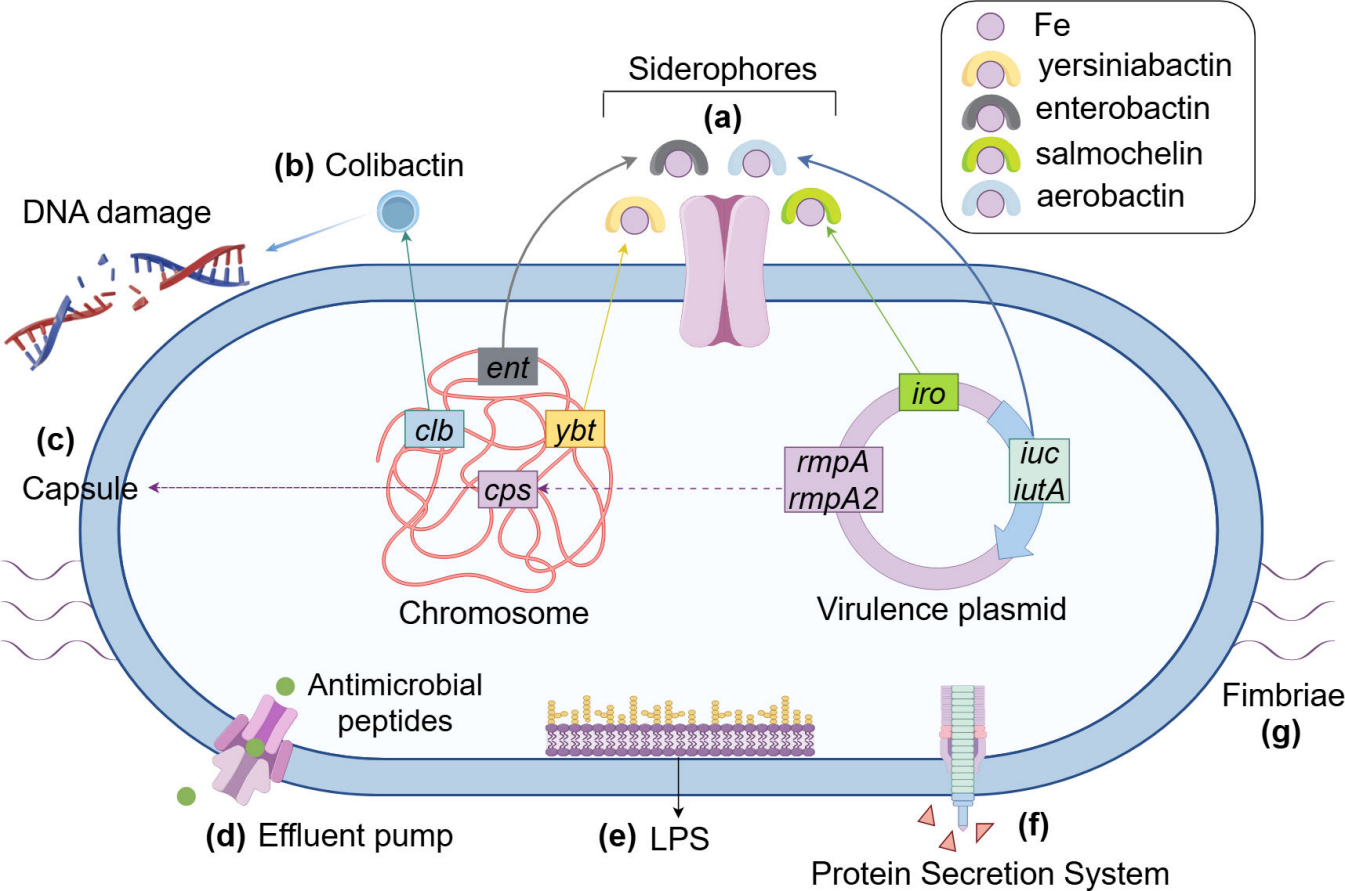
Virulence factors of hvKP. (**a**) Siderophores encoded by the virulence gene clusters *ent*, *ybt*, *iuc*, and *iro* on chromosomes and plasmids. (**b**) Colibactin can cause DNA damage in the host. (**c**) The *cps* and *rmpA/rmpA2* gene clusters mediate the formation of the mucus phenotype by regulating the synthesis of the capsule. (**d**) The efflux pump maintains and enhances pathogenicity by expelling antimicrobial peptides. (**e**) LPS on cell membranes that regulate the immune response. (**f**) The protein secretion system interferes with the survival of host cells and surrounding bacteria by secreting toxic effector proteins. (**g**) Fimbriae on the surface of bacteria enhance bacterial adhesion (the figure is produced by Figdraw).

cKP is the most common type and is closely associated with hospital-acquired infections. It occurs mainly in patients with chronic underlying diseases or who are immunocompromised (2). hvKP usually causes community-acquired infections such as pyogenic liver abscess, endophthalmitis, and meningitis in young healthy people (3). The high pathogenicity of hvKP is demonstrated by its invasiveness and its confrontation with the host immune system, which are dependent on various virulence factors on the strain (Fig. 1). Currently, the critical virulence factors considered for *K. pneumoniae* include the capsule, lipopolysaccharide (LPS), fimbriae, and siderophores (4). In addition, recent studies have shown that colibactin, the efflux pump AcrAB, and some secretion systems are also involved in the virulence of *K. pneumoniae* (5–7). With the widespread use of antibiotics in clinical practice, the problem of drug resistance in *K. pneumoniae* has become increasingly serious, and MDR or even extremely drug-resistant (XDR) strains have emerged. Carbapenem resistance is most frequently of concern, and carbapenem-resistant Enterobacteriaceae (CRE) remain a critical group in the latest list of bacterial priority pathogens published by the World Health Organization (WHO), with carbapenem-resistant *K. pneumoniae* (CRKP) being the clinically predominant CRE (8). Now CRKP strains have appeared in all WHO regions, some of which have a prevalence of 60%. The major global clonal group (CG) of CRKP is CG258, of which sequence type (ST) 11 is mainly distributed in China and South America, while ST258 is the predominant CRKP strain in the United States and some European countries (9, 10). For a long time, the hypervirulent and MDR phenotypes of *K. pneumoniae* were hardly compatible; however, with the interaction between virulence factors and resistance genes, a large number of reports of carbapenem-resistant hypervirulent *K. pneumoniae* (CR-hvKP) emerged, which posed great challenges to clinical anti-infective therapy (11). These changes reflect the complexity and adaptability of bacterial evolution, in which mobile genetic elements (MGEs) play important roles in the formation and spread of the above strains.

**Fig 2:**
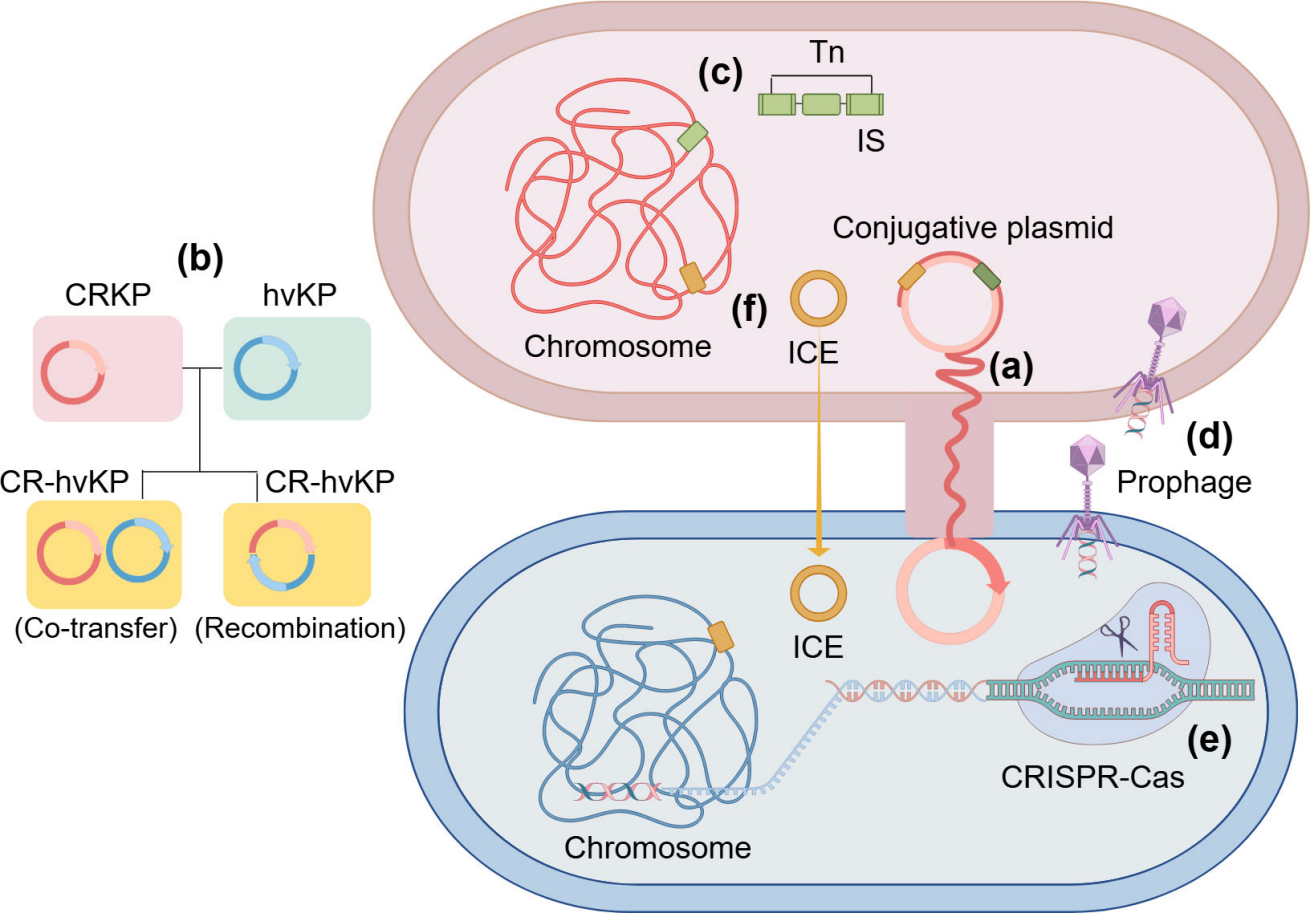
MGEs in *K. pneumoniae* and their transmission mechanisms. (**a**) Conjugative plasmid moves between bacteria by conjugative transfer. (**b**) The evolutionary mechanisms of CR-hvKP include the transfer or recombination of resistance plasmids and virulence plasmids. (**c**) IS and Tn move into bacterial chromosome and plasmid by encoding transposases. (**d**) Prophages integrate their own genome into bacterial chromosome and replicate with host DNA. (**e**) Bacteria recognize and cleave foreign DNA through the CRISPR-Cas system to resist plasmid and phage invasion. (**f**) Integrative conjugative element (ICE) integrates into the host genome by conjugation (the figure is produced by Figdraw).

MGEs are DNA fragments, such as plasmids, prophages, transposons, and insertion sequences (IS), that are able to move within the bacterial genome or between different bacteria (12). Bacteria acquire exogenous genes via horizontal gene transfer (HGT) through various MGEs and integrate them into their own genome, thereby acquiring a new phenotype (Fig. 2). Currently, many studies are focused on the role of MGE-mediated HGT in bacterial genome evolution and adaptation to environmental stresses (13, 14). In this paper, we review the common types of MGEs and their characteristics in *K. pneumoniae* and emphasize the critical role of MGEs in bacterial evolution, aiming to provide a theoretical basis for identifying new drug targets and innovative therapeutic strategies.

## PLASMID

Plasmids are circular double-stranded DNA molecules that exist independently of chromosomes and can replicate autonomously, with sizes ranging from 1 to 300 kb. On the basis of the integrity of the conjugation transfer module and the differences in transferability, plasmids can be classified into conjugative plasmids, mobilizable plasmids, and nonmobilizable plasmids (15). Conjugative plasmids possess transfer functional modules such as the origin of the transfer site (oriT), relaxase, type IV coupling protein, and type IV secretion system, thus enabling them to move between cells via conjugative transfer. Mobilizable plasmids contain at least the oriT and relaxase genes and require the assistance of other conjugative elements to achieve transfer. Nonmobilizable plasmids are unable to be transferred due to the lack of oriT, but they can be disseminated by means of transformation or transduction (16, 17). As a key component of MGEs, plasmids typically carry genes that encode specific functions, including antibiotic resistance and virulence genes, enabling bacteria to survive in complex host environments (18).

### Plasmid-influenced antibiotic resistance

Plasmids play a critical role in the acquisition and spread of antibiotic resistance. Research indicates that common antibiotic resistance genes (ARGs) of *K. pneumoniae* are often located on plasmids and can achieve intra- and interspecies spread of resistance through plasmid-mediated HGT (19–21). Plasmids carrying genes encoding carbapenemases are often of great concern because the emergence of carbapenemases has significantly increased the difficulty of treating multidrug-resistant Gram-negative infections and poses a serious threat to public health. The earliest carbapenemase IMP-1 was detected in a *K. pneumoniae* strain from Japan in 1991. Other species of carbapenemases, such as New Delhi metallo-β-lactamase (NDM), Verona intergron-encoded metallo-β-lactamase (VIM), and OXA-48, were subsequently also identified, but *K. pneumoniae* carbapenemase (KPC) is the most common and influential in *K. pneumoniae* (22). HGT of plasmid-borne *bla*_KPC_ is associated with the incompatibility (Inc) group. The plasmid pKpQIL was the first plasmid to be identified carrying *bla*_KPC_, which was isolated from the ST258 CRKP strain in Israel in 2006; subsequently, this plasmid and its derivatives have been reported in multiple countries, further confirming its epidemic (23). The pKpQIL is 113,637 bp in size and belongs to the IncFIIK2 group, which consists of a large backbone of the pKPN4-like plasmid and carries a transposable element containing *bla*_KPC-3_ (24). Unlike pKpOIL, in non-ST258 *K. pneumoniae* strains, the *bla*_KPC_ gene is present in the NTEKPC element of the plasmid, such as pKp048, an IncFIIK5 plasmid carrying *bla*_KPC-2_, which is widespread in China and most commonly associated with ST11 strains (25). In addition to IncFII, the *bla*_KPC_ gene is also detected on other types of plasmids, such as IncR, IncN, and IncX3, which exhibit a broad host range and are able to replicate and move to other Enterobacteriaceae by conjugative transfer (26, 27), and plasmids carrying *bla*_KPC_ usually contain multiple ARGs to other drugs, such as β-lactams, aminoglycosides, macrolides, tetracyclines, and quinolones, further exacerbating the antibiotic resistance crisis because obtaining these circulating plasmids converts bacteria into MDR or XDR strains (18).

Ceftazidime/avibactam (CAZ/AVI), tigecycline, and colistin are considered the last line of clinical treatment for CRKP infections, but unfortunately, resistant strains inevitably emerge as the use of these antibiotics increases (28, 29). CAZ/AVI is a novel combination of β-lactam/β-lactamase inhibitors that has good antibacterial activity against carbapenemase-producing *K. pneumoniae*, including Ambler class A enzymes and some class C and D enzymes (e.g., KPC and OXA-48), but it has no activity against class B enzymes such as NDM, so acquisition of plasmids carrying the metallo-β-lactamase gene would result in CAZ/AVI resistance (30). The study reported that the CRKP strain exhibited resistance to CAZ/AVI during treatment, and it contains an additional IncX3 plasmid carrying *bla*_NDM-5_ compared to earlier susceptible isolates (31). Tang et al. (32) found that the IncX3_NDM-5 plasmid showed high stability and transmissibility in *E. coli*, and the conjugation frequency remained at 10 ^−3^ to 10^−5^ with increasing CAZ/AVI concentration. Colistin and tigecycline resistance mechanisms are often associated with chromosomal mutation-mediated modification of lipid A and overexpression of efflux pump (33); however, plasmid-mediated HGT also plays an important role. Horizontal transfer of plasmid-borne *mcr* promotes the spread of colistin resistance from animal- to human-derived Enterobacteriaceae strains (34). Currently, a total of three plasmid-borne *mcr* genes have been found in *K. pneumoniae*, including *mcr-1*, *mcr-7*, and *mcr-8* (35). The *mcr* encodes a phosphoethanolamine (pEtN) transferase that adds pEtN to lipid A, altering the charge and structure of lipid A, which reduces the affinity of colistin for lipid A, leading to colistin resistance (36). Plasmid-borne variants of the tigecycline resistance gene *tet*(X) mediate high levels of resistance to tigecycline in *K. pneumoniae* (37). In addition, a novel plasmid-encoded resistance nodulation division efflux pump gene cluster, *tmexCD1-toprJ1*, was reported to reduce tigecycline sensitivity (38). Worryingly, recent studies (39, 40) have found that colistin resistance genes and tigecycline resistance genes coexist on the same strain, conferring transferable multidrug resistance in *K. pneumoniae*. These findings highlight that resistance plasmids pose a great threat to the clinical use of antibiotics and therefore warrant continued attention and surveillance.

### Plasmid-influenced virulence factors

Plasmids are not only vectors for resistance genes but also affect the formation of the virulence phenotype of hvKP by carrying multiple virulence genes. The hvKP strain was first reported in Taiwan, China, in 1986, and infected patients presented with pyogenic liver abscesses without biliary tract disease (41), followed by similar cases in some western countries (42, 43). In the same year, Nassif et al. (44) found that the deletion of the plasmid pKP100 encoding aerobactin resulted in the loss of virulence in *K. pneumoniae* K2 isolate, preliminarily confirming that the presence of the plasmid correlates with the virulence phenotype. In *K. pneumoniae*, the most intensively studied virulence plasmids are the plasmid pLVPK (45) isolated from strain CG43 (serotype K2, ST86 type) and the plasmid pK2044 (46) from strain NTUH-K2044 (serotype K1, ST23 type), which carry gene clusters with high homology and identity, including mucus regulators *rmpA* and *rmpA2*, siderophore genes *iucABCDiutA* and *iroBCDN*, and certain heavy metal resistance genes (47, 48). Struve et al. (49) found that pLVPK-like virulence plasmids were detected in all 30 hvKP strains isolated from different countries between 1996 and 2012. Similarly, a comparative analysis of the genome of hvKP CG23 by Lam et al. revealed that 94 of 97 strains carried the pK2044-like plasmid backbone, although some plasmids had deletion mutations at virulence sites (50). Molecular markers *peg-344* (encoding metabolite transporters), *iroB*, *iucA*, *rmpA*, and *rmpA2* on virulence plasmids have been shown to distinguish hvKp from cKP with diagnostic accuracy >0.95 (51).

The virulence phenotype of *K. pneumoniae* is partly attributed to genes encoding virulence factors on plasmids. Capsular polysaccharide is a layer of polysaccharide covering the outside of bacteria and plays an important role in bacterial pathogenicity and immune escape (2). Its synthetic gene is located in the *cps* gene cluster on the bacterial chromosome, while the regulatory genes *rmpA* and *rmpA2* located on the plasmid can increase the capsule thickness by up-regulating the transcription of synthetic genes, so hvKP shows the hypermucoviscosity (HMV) phenotype and enhances its anti-phagocytic ability (52). Siderophores are important virulence factors secreted by *K. pneumoniae* that help bacteria acquire iron ions in an iron-limited environment. Research results indicate that, compared with cKP, hvKP can secrete greater quantities and more active siderophores (53). There are four siderophores in *K. pneumoniae*, including aerobactin, salmochellin, yersiniabactin, and enterobactin. Among them, the synthetic sites of aerobactin and salmochellin are located in the *iucABCDiutA* and *iroBCDN* gene clusters of virulence plasmids, respectively, while yersiniabactin and enterobactin are usually encoded by the *ybt* and *ent* gene clusters on chromosomes (54). In addition, a new Klebsiella ferric uptake transporter, *kfu*, has been discovered and is also considered a potential virulence factor (55). Plasmids carrying antibiotic resistance genes have also been found to harbor genes involved in regulating capsule synthesis, biofilm formation, and type 3 fimbrial expression, thereby enhancing the virulence of *K. pneumoniae* (56, 57). In conclusion, the synergistic effect of multiple virulence genes on plasmids enhances the virulence of *K. pneumoniae*. Therefore, enhancing the identification of virulence phenotypes and developing targeted inhibitors of virulence factors could effectively reduce the pathogenicity of *K. pneumoniae*.

### Evolution of CR-hvKP

CR-hvKP is a novel hospital-acquired pathogen, and several outbreaks have been reported in Asian regions (58, 59). Such strains show characteristics of high virulence, multidrug resistance, and high transmissibility, so CR-hvKP is regarded as a “superbug” threatening human health (11). The evolutionary pathways of CR-hvKP involve the acquisition of virulence plasmids by CRKP, the transfer of carbapenem resistance genes to hvKP, and the acquisition of hybrid plasmids carrying virulence and carbapenem resistance genes by cKP (60–62).

Classic virulence plasmids such as pLVPK (45) are generally considered nonconjugative plasmids because they lack the *tra* gene cluster encoding conjugative transfer modules. However, they can be transferred to new hosts with the assistance of other conjugative transfer elements or by fusion with conjugative resistance plasmids (63). Yang et al. (64) found that plasmid p15WZ-82_Vir was formed by integrating a 100 kb fragment of the virulence plasmid pLVPK into the conjugative IncFIB plasmid This resulting plasmid had conjugative transfer and enabled CRKP strains to express both hypervirulence and carbapenem resistance phenotypes. Xie et al. (65) identified a novel IncFIA plasmid that could be fused with a hypervirulent plasmid by homologous recombination to form a hybrid conjugative plasmid. Results of conjugation experiments showed that this hybrid plasmid allowed cKP and CRKP to express the hypervirulent phenotype. Wang et al. (66) reported a self-transferable IncN3 plasmid with a high conjugation frequency, which can mobilize co-existing nonconjugative virulence or resistance plasmids to CRKP or hvKP, respectively. In addition, the lncN3 plasmid can also be fused with virulence or resistance plasmids through a replicative transposition mechanism to achieve the evolution of CR-hvKP. In fact, horizontal transfer of plasmids does not occur independently and usually involves complex interactions with other MGEs, and frequent transposition events lead to plasmid fusion or recombination, thereby better adapting to environmental stresses and host conditions. It usually involves complex interactions with other MGEs, enabling bacteria to engage in frequent transposition events, leading to plasmid fusion or recombination, thereby better adapting to different environmental stresses and host conditions (67). Thus, the continued emergence and mechanistic diversity of plasmid-mediated HGT await further investigation.

## INSERTION SEQUENCE AND TRANSPOSONS

The IS is the simplest transposable element and generally does not carry any genes unrelated to transposition function except transposase genes (68). IS can exist independently or be part of a transposon, and its effects on the genome are as follows: IS can insert upstream of the genes and regulate the expression of neighboring genes through its own promoter or forming a hybrid promoter. Subsequently, the IS can be used as a vector for genes to promote the transmission of virulence and drug resistance (69, 70). ISs are prevalent on conjugative plasmids and are dynamically distributed in high abundance. Che et al. (71) confirmed that 63.2% of ARG transfers between plasmids and chromosomes was attributed to ISs. It is worth noting that the IS6 family, especially IS26, plays a critical role in the accumulation and spread of multidrug resistance genes in gram-negative bacteria (72, 73). Porins are channel proteins on the outer membrane of bacteria that allow many small molecules to enter and exit cells, including hydrophilic antibiotics. Studies (74–76) have found that IS26 and IS5 interrupt the *ompK36* gene, resulting in loss of porin expression and increased carbapenem resistance in *K. pneumoniae*. IS-mediated *mrgB* disruption is the most widely reported mechanism of colistin resistance in *K. pneumoniae* (77, 78). The *mgrB* is a negative regulator of the two-component system PhoPQ, and when it is inactivated, overactivation of PhoPQ triggers an increase in lipid A modification leading to colistin resistance and enhanced bacterial virulence (79). One of the major insertion sequences is IS*Kpn26*, and *K. pneumoniae* carrying IS*Kpn26* increases the risk of polymyxin treatment failure (80, 81). Wang et al. (82) found that IS*Kpn26*-induced *wcaJ* inactivation reduced capsule synthesis, thereby impairing hvKP virulence; however, isolates with *wcaJ* disruptions exhibited a lower fitness cost and higher conjugation frequency to the *bla*_KPC-2_ plasmid, suggesting that *wcaJ* has a potential role in promoting CR-hvKP formation.

Transposons (Tns) are a class of DNA sequences that can replicate and move autonomously in the genome, carrying the ISs at both ends, and functional genes including drug resistance genes, heavy metal resistance genes, and virulence genes in the central region (83). The genes carried by Tn can follow the activity of Tn, resulting in gene rearrangement, gene mutation, or changes in gene expression near the insertion site. The Tn3 family (84) was the first identified bacterial transposon and is often associated with the spread of antibiotic resistance. In particular, the mobile transposon Tn4401 is able to mobilize the *bla*_KPC_ gene at a high frequency and is considered to be the origin of *bla*_KPC_-like gene acquisition and transmission (85). In addition, the Tn3 transposon is involved in the formation and spread of CR-hvKP strains. Tian et al. (86) demonstrated that the virulence plasmid pVir could be fused with the conjugative plasmid pKPC by Tn3-mediated homologous recombination, thereby achieving co-transfer of drug resistance and virulence. In conclusion, ISs and Tns are widespread in bacterial genomes, and their activities significantly influence gene expression and genetic stability.

## PROPHAGE

Prophage is a dormant state of bacteriophage DNA that can integrate into the bacterial chromosome and replicate along with the host genome, and bacteria with phage genomes are known as lysogenic bacteria. Prophages can impact essential biological properties of their host bacteria, including virulence, biofilm formation, and fitness (87). Under certain stress conditions, prophages can be excised from the host genome and enter the lytic cycle. At this point, phage genes begin to be expressed, releasing phage particles and leading to lysis and death of the host bacteria.

*K. pneumoniae* contains abundant prophages, dominated by the *Myoviridae* family with relatively small genomes, followed by the *Siphoviridae* family (88). The prophages integrated on plasmids and chromosomes have different characteristics. The number of intact prophages on chromosomes was much greater, whereas prophages on plasmids are mostly incomplete, and a high proportion of defective prophages may be associated with the conjugative transfer of plasmids. Furthermore, prophages integrated on chromosomes predominantly affect the virulence of strains, and prophages on plasmids play a major role in antibiotic resistance of strains (89). Wang et al. (90) analyzed 20 prophage DNA sequences from the *sap* sites of *K. pneumoniae* chromosomes, revealing varying degrees of base loss and gain in the backbone region near the integration sites. This widespread genetic variation confers diversity in prophage DNA evolution and enhances the adaptability of host bacteria to environmental stress.

In the context of increasing antibiotic resistance, phage therapy has gradually attracted attention (91, 92). Phage depolymerases can specifically recognize and bind to capsular polysaccharides and then degrade them through hydrolysis, enabling phages to break through the barrier of the capsule and achieve infection and lysis of bacteria (93). Currently, more than 40 specific polysaccharide depolymerases have been characterized in *K. pneumoniae*, and some of which can significantly enhance the bactericidal effect against hvKP (94). These findings suggest that phages and polysaccharide depolymerases are antimicrobial agents with great potential, but the safety and stability of their use in practical applications need to be further confirmed (95).

## CRISPR-CAS SYSTEM

Clustered regularly interspaced short palindromic repeats (CRISPR) constitute an acquired immune system found in various bacteria and archaea. It functions by utilizing Cas proteins to degrade target genes, thereby resisting invasion from foreign viruses or plasmids and regulating microbial behavior and pathogenicity (96). CRISPR-Cas systems in *K. pneumoniae* mainly include types I–E and subtypes I–E (I–E*) systems, and their distributions are associated with multilocus sequence typing (MLST) and pulsed-field gel electrophoresis (97). CRISPR is naturally antagonistic to phages and is an important means of inhibiting the invasion of MGEs. It has been shown that the presence of the CRISPR-Cas system is negatively associated with antibiotic resistance and can make it more difficult for *K. pneumoniae* to acquire exogenous resistance genes (98), especially the types I–E CRISPR-Cas system, which can effectively inhibit the transmission of resistance mediated by the IncF plasmid (99, 100). Zhou et al. (101) used endogenous CRISPR-Cas3 to mediate IncFII plasmid curing to restore susceptibility to multiple antibiotics in drug-resistant *K. pneumoniae*, demonstrating the great potential of gene editing technology.

CRISPR-Cas systems provide new targets and strategies to address the problem of bacterial resistance, but the emergence of anti-CRISPR proteins (Acr) poses great challenges to the application of this system. Acr is a phage-encoded regulatory protein that can antagonize the activity of CRISPR-Cas systems; it was first reported in *Pseudomonas aeruginosa* in 2013 (102), and since then, many types of Acr proteins have been successively discovered (103). AcrIE8 is the first Acr protein identified in the prophage of *K. pneumoniae* to inhibit the type I-E CRISPR systems (104). In addition, a study reported that a novel Acr protein, AcrIE9.2, is prevalent in ST15-type CRKP strains; AcrIE9.2 can inhibit I–E*-type CRISPR-Cas systems and is associated with the propagation of the bla_KPC_ plasmid (105), and its specific mechanism and regulatory role need to be further investigated. The existence of the CRISPR-Cas systems and the Acr protein reflects the mutual constraints and coevolution between the bacterial defense system and phages. Acr proteins are known as “switches” of the CRISPR-Cas systems, and their diversity and complex mechanisms of action have great potential in gene editing and gene regulation (106).

## INTEGRATIVE CONJUGATIVE ELEMENT

Integrative conjugative elements (ICEs), also known as conjugative transposons, are a class of mobile genetic elements with integrated excision and transfer capabilities. The integrases in ICE can recognize specific sites on the host chromosomes and integrate their own DNA into the host genome through recombination mechanisms (107). When stimulated by certain signals, ICE can excise from the host chromosome to form independent circular DNA, which is then transferred from the donor bacterium to the recipient bacterium through a conjugation device (type IV secretion system). ICEs have a highly modular molecular structure, including recombination, conjugation, regulation, and other parts, which affect the phenotype and adaptability of strains by carrying various cargo genes encoding virulence, resistance, and metabolism (108).

ICE*Kp* is an important virulence genetic element that significantly impacts the pathogenicity of *K. pneumoniae*. Lam et al. (109) found that 97% of ICE*Kp* sequences were present in *K. pneumoniae* by phylogenetic and structural comparison, and a few other Enterobacteriaceae occasionally acquired ICE*Kp*. The results of the whole-genome sequence alignment showed that ICE*Kp* usually carried the *ybt* gene cluster, and the structural classification of ICE*Kp* had a good correspondence with the *ybt* lineage. ICE*Kp1* was the first ICE reported in *K. pneumoniae*. In the genome of the hvKP strain NTUH-K2044, ICE*Kp1* integrates near the asparagine (Asn) tRNA loci on the chromosome; additionally, it inserts the pLVPK-like *iroNBCD* gene and the *rmpA* gene between the basal *ybt* and the integrated conjugation module to mediate salmonellin synthesis and the mucoid phenotype (109). Notably, ICE*Kp* may have a selective distribution in high-risk clones of *K. pneumoniae*. Farzand et al. (110) reported that 81.6% of ST258 strains carried ICE*Kp2*. Unlike ICE*Kp1*, which has variable cargo genes, the cargo genes of ICE*Kp2* are highly conserved with little sequence diversity. However, the conjugative protein Mob2 of ICE*Kp2* may enhance the efficiency of plasmid transfer driven by coexisting ICE*Kp1* in the strain, suggesting that there is an interaction between different ICEs. ICE*Kp10* appears to be the most prevalent ICE in CG23 hvKP (50, 111), and its structure is similar to the KPHPI208 genomic island (112) in the liver abscess strain 1,084. ICE*Kp10* is typically characterized by carrying the *clb* gene cluster encoding colibactin. Colibactin is an important virulence factor in *K. pneumoniae* that causes host DNA double-strand breaks and disrupts host immune defense mechanisms (113). In addition, colibactin may help hvKP to kill the commensal bacteria, thus better competing for living space and nutrients in the complex gut microbiota (6). Among the ST11-type CRKP, ICE*Kp3* is more prevalent, suggesting that the cargo genes on ICEKp3 may play a key role in the drug resistance of such strains (114). In conclusion, ICEs are widely distributed in the *K. pneumoniae* genome, and the mechanism of these genetic elements in the transmission of hypervirulent and MDR bacteria awaits in-depth study.

## CONCLUSION AND PERSPECTIVE

MGEs have a profound effect on the genetic diversity and adaptive capacity of bacteria. This review discusses the important MGEs that influence the resistance, virulence, and environmental adaptability of *K. pneumoniae*, focusing on the central role of plasmids, especially conjugative plasmids, in MGEs. Because plasmids have unique replication and transfer mechanisms, they are able to spread independently between bacteria through conjugative transfer, whereas other MGEs, such as Tns and ICEs, need to be integrated on plasmids or chromosomes to achieve transfer. In addition, CR-hvKP has spread widely in China but lacks effective treatments. The formation of such strains is largely attributed to the transfer of conjugative plasmids carrying resistance or virulence genes, as well as the fusion or recombination of plasmids. Therefore, studying plasmids and their transmission mechanisms is key to coping with these “superbugs.”

There is a wide variety of MGEs in *K. pneumoniae*, and only some of the common types are listed in this review. Thanks to the innovation of sequencing technologies and the wide application of bioinformatics, we can obtain relevant information about MGEs through a variety of databases and software (Table 1), which is not only helpful to understand the function and evolutionary relationships of MGEs but also helpful to explore pathogenesis and drug resistance development of bacteria. In the future, the identification and surveillance of MGEs should be strengthened to prevent outbreaks and epidemics in hospitals.

**TABLE 1:** MGEs analysis tools

Software or database	Function	Website	Reference	Classification
plasmidfinder	Identification of the replicon gene of the plasmid genome and the corresponding lnc type	https://cge.food.dtu.dk/services/PlasmidFinder/	(115)	Plasmid related
PubMLST	Detection and characterization of plasmid sequences in whole-genome sequencing data from Enterobacteriaceae	http://pubmlst.org	(116)	
PLSDB	An interactive view of all obtained plasmids with additional meta information	https://www.ccb.uni-saarland.de/plsdb	(117)	
oriTfinder	Identification of the origin of transfer site (oriT) of a conjugative plasmid or chromosome-borne integrative and conjugative element	http://bioinfo-mml.sjtu.edu.cn/oriTfinder	(118)	
ISfinder	Prediction and classification of ISs on the genome	https://isfinder.biotoul.fr/about.php	(119)	IS related
Transposon registry	Assignment of Tn numbers for new bacterial and archaeal elements and provision of a searchable repository for all transposons in the genome	http://transposon.lstmed.ac.uk/	(120)	Tn related
HTT-DB	Storage and analysis of transposon horizontal transfer events in different species	http://lpa.saogabriel.unipampa.edu.br:8080/httdatabase	(121)	
PHASTER	Identification and annotation of phage sequences	http://phaster.ca/	(122)	Phage related
CRISPRCasFinder	Identification of both CRISPR arrays and Cas proteins	https://crisprcas.i2bc.paris-saclay.fr	(123)	CRISPRCas related
ICEberg	Prediction of ICE components	http://db-mml.sjtu.edu.cn/ICEberg/	(124)	ICE related
ICEfinder	Detection of ICEs on bacterial genomes	https://bioinfo-mml.sjtu.edu.cn/ICEfinder/ICEfinder.htm	(125)	
VRprofile2	Detection of antibiotic resistance-associated mobilome in bacterial pathogens	https://tool2-mml.sjtu.edu.cn/VRprofile	(126)	MGEs related
MGEFinder	Identification of MGEs inserted and integrated into the genome	http://github.com/bhattlab/MGEfinder	(127)	
ACLAME	Collection and classification of MGEs from various sources	http://aclame.ulb.ac.be	(128)	

## AFTER EPUB

[This article was published on 29 April 2025 with missing funding information. The funding information was updated in the current version, posted on 6 May 2025.]
